# Effect of small molecule eRF3 degraders on premature termination codon readthrough

**DOI:** 10.1093/nar/gkab194

**Published:** 2021-03-25

**Authors:** Alireza Baradaran-Heravi, Aruna D Balgi, Sara Hosseini-Farahabadi, Kunho Choi, Cristina Has, Michel Roberge

**Affiliations:** Department of Biochemistry and Molecular Biology, University of British Columbia, Vancouver, BC V6T 1Z3, Canada; Department of Biochemistry and Molecular Biology, University of British Columbia, Vancouver, BC V6T 1Z3, Canada; Department of Biochemistry and Molecular Biology, University of British Columbia, Vancouver, BC V6T 1Z3, Canada; Department of Biochemistry and Molecular Biology, University of British Columbia, Vancouver, BC V6T 1Z3, Canada; Department of Dermatology, Medical Center-University of Freiburg, Faculty of Medicine, Freiburg, Germany; Department of Biochemistry and Molecular Biology, University of British Columbia, Vancouver, BC V6T 1Z3, Canada

## Abstract

Premature termination codon (PTC) readthrough is considered a potential treatment for genetic diseases caused by nonsense mutations. High concentrations of aminoglycosides induce low levels of PTC readthrough but also elicit severe toxicity. Identifying compounds that enhance PTC readthrough by aminoglycosides or reduce their toxicity is a continuing challenge. In humans, a binary complex of eukaryotic release factors 1 (eRF1) and 3 (eRF3a or eRF3b) mediates translation termination. They also participate in the SURF (SMG1-UPF1-eRF1-eRF3) complex assembly involved in nonsense-mediated mRNA decay (NMD). We show that PTC readthrough by aminoglycoside G418 is considerably enhanced by *eRF3a* and *eRF3b* siRNAs and cereblon E3 ligase modulators CC-885 and CC-90009, which induce proteasomal degradation of eRF3a and eRF3b. eRF3 degradation also reduces eRF1 levels and upregulates UPF1 and selectively stabilizes *TP53* transcripts bearing a nonsense mutation over WT, indicating NMD suppression. CC-90009 is considerably less toxic than CC-885 and it enhances PTC readthrough in combination with aminoglycosides in mucopolysaccharidosis type I-Hurler, late infantile neuronal ceroid lipofuscinosis, Duchenne muscular dystrophy and junctional epidermolysis bullosa patient-derived cells with nonsense mutations in the *IDUA*, *TPP1*, *DMD* and *COL17A1* genes, respectively. Combination of CC-90009 with aminoglycosides such as gentamicin or ELX-02 may have potential for PTC readthrough therapy.

## INTRODUCTION

Nonsense mutations introduce a premature termination codon (PTC) into mRNA, causing ribosomes to terminate translation prematurely instead of synthesizing full-length functional proteins. These mutations account for about 11% of all genetic lesions in patients with inherited diseases (1). Some small molecules bind ribosomes such that they can recognize PTCs as sense codons, enabling the synthesis of full-length protein, a process termed PTC readthrough.

Aminoglycosides and their derivatives are among the most potent PTC readthrough compounds found to date (2). They bind to the ribosomal decoding A site and induce a conformation change that enables pairing of a near-cognate aminoacyl-tRNA to the PTC and continued translation. However, low rates of PTC readthrough and *in vivo* toxicity makes it challenging to develop and use these aminoglycosides as life-long treatment in patients with genetic diseases (3,4). Recently, major efforts have been put into developing compounds that potentiate the PTC readthrough activity of aminoglycosides. Although several structurally unrelated compounds that enhance PTC readthrough have been identified (5–8), understanding their mechanism of action and reducing their toxicity is a continuing challenge.

In eukaryotes, translation termination is facilitated by a binary complex of eukaryotic release factors (eRF) 1 and 3. Recognition of the stop codons takes place at the ribosomal A site through direct interaction with eRF1. Binding of eRF1 to the stop codon triggers peptidyl-tRNA hydrolysis and release of the newly synthesized polypeptide (9,10). In mammals, two *eRF3* genes, *eRF3a*/*GSPT1* and *eRF3b*/*GSPT2*, encode eRF3a and eRF3b, also called GSPT1 and GSPT2, which are GTPases and can each bind eRF1 to stimulate its translation termination activity (11–13). In humans, eRF3a and eRF3b share almost 88% identity. *eRF3b* lacks the intronic sequences seen in *eRF3a* and the eRF3b N-terminal domain lacks the polyglycine tract present in eRF3a. According to the Human Protein Atlas, eRF3a is ubiquitously expressed in human cell lines originating from various organs while eRF3b expression was detected in only a subset of tested cell lines (14, www.proteinatlas.org). However, most human tissues express both eRF3a and eRF3b, with eRF3b levels generally being lower than those of eRF3a (14, www.proteinatlas.org). The translation termination factors are also involved in modulating nonsense-mediated mRNA decay (NMD) by participating in the SURF (SMG1–UPF1–eRF1–eRF3) complex (15). NMD is a post transcriptional surveillance mechanism that recognizes and degrades PTC-bearing mRNAs. The first step in NMD is the formation of the SURF complex on ribosomes stalled at PTCs, followed by phosphorylation and activation of the ATP-dependent RNA helicase UPF1.

Similar to yeast, where depletion of translation termination factors enhances nonsense suppression (16,17), it has been shown that small interfering RNA (siRNA) or antisense oligonucleotides (ASOs) targeting *ETF1*, encoding eRF1 or *eRF3a* promote PTC readthrough in reporter assays in human cells (18,19). Recently, it was also shown that ASOs targeting *Etf1* or *Gspt1* (mouse ortholog of human *eRF3a/GSPT1*) can promote PTC readthrough in a hemophilia mouse model with nonsense mutations, particularly in combination with the readthrough aminoglycoside G418 (20). Collectively, these studies illustrate the potential of targeting eRF3a for treatment of genetic diseases caused by nonsense mutations. Whether depletion of eRF3b also promotes nonsense suppression is yet to be reported. Also, the impact of concurrent depletion of all translation termination factors on PTC readthrough has not been determined. However, the development of RNA therapeutics remains a considerable challenge (21).

Recently, targeted protein degradation approaches have identified the small molecules CC-885 and CC-90009 as eRF3a degraders with potent tumoricidal activity against acute myeloid leukemia (AML) cells (22–27). These small molecules are cereblon (CRBN) E3 ligase modulators that mediate binding of eRF3a to cereblon and induce the ubiquitination and proteasome-dependent degradation of eRF3a (22,24). Unlike CC-885, which triggers the degradation of additional CRBN substrates, CC-90009 seems to have little to no effect on the rest of the proteome and CC-90009 is the first eRF3a degrader to enter clinical trials in patients with relapsed or refractory AML (24,26).

In this study, we investigate the effect of small molecule eRF3a degraders on PTC readthrough in several genetic disease models. We show that CC-885 and CC-90009 reduce not only the levels of eRF3a, but also those of eRF3b and eRF1 and that they act synergistically with aminoglycosides to suppress NMD and increase PTC readthrough.

## MATERIALS AND METHODS

### Human cells

The HDQ-P1 cell line with homozygous nonsense mutation in the *TP53* gene (NM 000546.5:c.637C>T; NP 000537.3:p.R213X) was purchased from the German Collection of Microorganisms and Cell Cultures (DSMZ, Germany). The Caov-3 cell line with homozygous nonsense mutation in the *TP53* gene (NM 000546.5:c.406C>T; NP 000537.3:p.Q136X) and HCT116 cell line with wild-type *TP53* were purchased from the American Type Culture Collection. GM00798 primary fibroblasts from a patient with Hurler syndrome with a homozygous nonsense mutation in *IDUA* gene (NM_000203.5:c.1205G > A; NP_000194.2:p.W402X) and GM16485 primary fibroblasts from a patient with late infantile neuronal ceroid lipofuscinosis with compound heterozygous nonsense mutations in the *TPP1* gene (NM_000391.3:c.379C > T/c.622C > T; NP_000382.3:p.127X/p.R208X) were purchased from the Coriell Biorepository, USA. HDQ-P1, Caov-3, HCT116, GM00798 and GM16485 cells were cultured in high glucose Dulbecco's modified Eagle medium (DMEM, Sigma-Aldrich) supplemented with 10% (vol/vol) FBS (Sigma-Aldrich) and 1% antibiotic-antimycotic (Gibco/Thermo Fisher Scientific) at 37°C and 5% (vol/vol) CO_2_. SW900 cells with homozygous nonsense mutation in the *TP53* gene (NM 000546.5:c.499C>T; NP 000537.3:p.Q167X) and NCI-H1299 cells with homozygous deletion of the *TP53* gene (NM_000546.5:c.1–954>AAG; NP_000537.3:p.?) were purchased from the American Type Culture Collection and cultured in RPMI-1640 medium (Sigma-Aldrich) supplemented with 10% (vol/vol) FBS and 1% antibiotic-antimycotic at 37°C and 5% (vol/vol) CO_2_. The NCI-H1299 cell line expressing p53 R213X-TGA was generated as previously described (8). In brief, NCI-H1299 cells were transfected with pcDNA-6.2/V5-DEST vector expressing p53 R213X-TGA mutant using lipofectamine 2000 reagents and were subjected to blasticidin selection (Thermo Fisher Scientific). Individual clones that were resistant to 10 μg/ml blasticidin were selected and amplified. C25CI48 immortalized myoblasts derived from an unaffected individual and HSK001 immortalized myoblasts derived from a Duchenne muscular dystrophy patient with a nonsense mutation in the *DMD* gene (NM_004006.2:c.6103G > T; NP_003997.1:p.E2035X) were provided by Vincent Mouly, Myobank, Sorbonne Universités. They were cultured in skeletal muscle cell growth medium (PromoCell, Germany) supplemented with 20% (vol/vol) FBS and 1% antibiotic–antimycotic at 37°C and 5% (vol/vol) CO_2_. Myoblasts were differentiated into myotubes in differentiation medium consisting of high glucose DMEM supplemented with 10 μg/mL insulin (Sigma-Aldrich) and 1% antibiotic-antimycotic (Gibco/Thermo Fisher Scientific). Immortalized JEB01 keratinocytes were derived from a junctional epidermolysis bullosa (JEB) patient with a homozygous nonsense mutation in the *COL17A1* gene (NM_000494.4:c.2062C > T;NP_000485.3:p.R688X). The use of human samples for research was approved by the Ethics Committee of the University of Freiburg, Germany (EK-Freiburg: 215/15). After written informed consent, primary keratinocytes were isolated from a part of a skin biopsy obtained for diagnostic purposes using standard methods. Immortalisation was performed by lentiviral transduction with 0.5 μl HPV-16 E6/E7 gene expression construct (LV617-GVO-ABM) and 10 μl ViralPlus Transduction Enhancer (Applied Biological Materials Inc.). HaCaT cells, a spontaneously transformed keratinocyte cell line from adult human skin were used as controls. Keratinocytes were cultured in defined keratinocyte serum-free medium (K-SFM) supplemented with defined K-SFM growth supplement (Gibco/Thermo Fisher Scientific) and 1% antibiotic–antimycotic (Gibco/Thermo Fisher Scientific) at 37°C and 5% (vol/vol) CO_2_. Medium was replaced every 2–3 days.

### Transient transfections

Knockdown of *eRF3a* and *eRF3b* was performed by reverse transfection of HDQ-P1 and NCI-H1299-p53 R213X cells using ON-TARGETplus Human *eRF3a* and *eRF3b* siRNAs (Dharmacon, a Horizon Discovery Group company). Transfection complexes containing 50 nM ON-TARGETplus siRNA and 3–4 μl lipofectamine 2000 (Thermo Fisher Scientific) in 50 μl Opti-MEM I (Thermo Fisher Scientific) were plated in 12-well tissue culture plates and 750 μl of cells at 2 × 10^5^ cells/ml was added to each well. Twenty-four hours later wells were replenished with fresh medium. Forty-eight hours after transfection, each sample was split into two wells of a 12-well plate and left either untreated or treated with 20 μg/ml G418. After 48 h, cells were lysed and subjected to automated capillary electrophoresis western analysis. For eRF3a and eRF3b overexpression, HDQ-P1 cells were transiently transfected with pcDNA3.1 vector expressing FLAG-tagged full-length eRF3a, eRF3a-G575N or eRF3b (GENEWIZ) using Lipofectamine 2000 (Thermo Fisher Scientific). Forty-eight hours after transfection, samples were split into 12-well tissue culture plates and 24 h later either left untreated or exposed to various concentrations of CC-885 or CC-90009 with or without 20 μg/ml G418. After 48 h, the cells were lysed and subjected to automated capillary electrophoresis western analysis.

### Automated capillary electrophoresis western analysis

The western analysis assays for eRF3a, eRF3b, eRF1, UPF1, SMG1, GAPDH, Vinculin, p53, Dystrophin and TPP1 detection were performed as previously described (8). Briefly, mixtures of cell lysates (0.1–1 mg/ml) and the fluorescent master mix were heated either at 95°C (p53, Dystrophin, TPP1, eRF1, UPF1, SMG1, Vinculin, GAPDH) or 37°C (eRF3a, eRF3b) for 5 min. The samples, blocking and chemiluminescent reagents, primary and secondary antibodies, and wash buffer were dispensed into the microplates and capillary electrophoresis western analysis was carried out with the ProteinSimple WES instrument. DO-1 mouse anti-p53 antibody (1:400, Santa Cruz sc-126), mouse anti-vinculin antibody (1:600, R&D Systems MAB6896), rabbit anti-eRF3a (1:100, Thermo Fisher Scientific PA5–62621), rabbit anti-eRF3b (1:100, Thermo Fisher Scientific PA5–60824), mouse anti-FLAG M2 (1:100, Sigma F1804), rabbit anti-GAPDH (1:800, Abcam ab128915), mouse anti-TPP1 (1:1000, Abcam ab54685), rabbit anti-Dystrophin (1:400, Abcam ab15277), rabbit anti-UPF1 (1:1000, Abcam ab109363), rabbit anti-SMG1 (1:50, Cell signaling 4993) and mouse anti-eRF1 (1:100, Novus Biologicals 4F9H12) were used. The data were acquired and analysed using the inbuilt Compass software (ProteinSimple) with the high dynamic range detection profile, which uses multiple substrate injections and exposure times. Electropherograms were converted to pseudo-blots and presented in figures for ease of visualization.

### SDS-PAGE and immunoblotting

Human JEB01 keratinocytes were seeded into 6 well tissue culture dishes (2 × 10^5^ cells/ 2 ml/well) and treated with various concentrations of CC-885 and CC-90009 with or without gentamicin for 72 h. After cell lysis, 15 μg total protein from each lysate was separated on a 6% polyacrylamide gel. Gels were electrotransferred onto a nitrocellulose membrane and blocked in Tris-buffered saline containing 0.1% (v/v) Tween 20 (TBS-T) supplemented with 5% (w/v) non-fat dry milk. Membranes were then incubated with rabbit anti-Collagen XVII antibody (1:1000, Abcam ab184996) overnight at 4°C. Membranes were washed three times with TBS-T and incubated with HRP-conjugated goat anti-rabbit secondary antibody and developed using enhanced chemiluminescence substrate (Millipore). After stripping using 0.1 N NaOH, membranes were reprobed with rabbit anti-beta actin antibody (1:10 000, Novus Biologicals) and detected as above.

### TPP1 enzyme activity assay

GM16485 primary fibroblasts seeded into 60 mm tissue culture dishes (5 × 10^5^ cells/ 3 ml/ well) were exposed to various concentrations of CC-885 and CC-90009 with or without 20 μg/ml G418. After 72 h cells were lysed and TPP1 enzyme activity was determined as previously described with minor modifications (8). In brief, cell lysates were diluted 1:5 in 50 mM sodium acetate pH 4.0 and pre-incubated at 37°C for 2 h. Then, three replicates of 20 μg protein of each lysate were incubated in 150 μl of 50 mM sodium acetate pH 4.0 containing a final concentration of 62.5 μM Ala-Ala-Phe-7-amido-4-methylcoumarin (Sigma A3401) for 2 h at 37°C. Fluorescence was measured using a TECAN Infinite M200 plate reader at excitation/emission wavelengths of 360/460 nm. TPP1 enzyme activity of lysate from unaffected fibroblasts was also determined in the same way and used as reference.

### Iduronidase activity

GM00798 primary fibroblasts seeded into 60 mm tissue culture dishes (5 × 10^5^ cells/3 ml/well) were exposed to various concentrations of CC-885 and CC-90009 with or without 20 μg/ml G418. After 72 h cells were lysed and iduronidase activity was determined as previously described (28). In brief, three replicates of 25 μl of each lysate were mixed with 25 μl of sodium formate buffer (0.4M sodium formate, 0.4M formic acid, pH 3.5) containing a final concentration of 25 μM 4-methylumbelliferyl α-L-iduronide (Glycosynth) and incubated at 37°C for 1h. Reaction stopped with addition of 200 μl 0.5M glycine–NaOH buffer pH10.3. Fluorescence was measured using a TECAN Infinite M200 plate reader with excitation/emission wavelengths of 365/450 nm. Iduronidase activity of lysate from unaffected fibroblasts was also determined in the same way and used as reference.

### Cell viability assay

HDQ-P1 cells, WT and GM00798 fibroblasts, and C25CI48 myoblasts were seeded at 5000 cells/well in 96-well plates in triplicate. The next day cells were exposed to various concentrations of CC-885 or CC-90009 compounds with or without 20 μg/ml G418. HaCaT keratinocytes were seeded at 20,000 cells/well and treated with CC-885 or CC-90009 compounds with or without 100 μg/ml gentamicin. After 48 h, cell viability was determined by MTT assay as previously described (29). In brief, 25 μl of a 5 mg/ml solution of 3-(4,5-dimethylthiazol-2-yl)-2,5-diphenyltetrazolium bromide (Sigma) in phosphate-buffered saline was added to cells in the presence of 100 μl of cell culture medium and incubated at 37 °C. After 2h, 100 μl of extraction buffer (20% sodium dodecyl sulfate dissolved in dimethylformamide/water (1:1), pH 4.7) was added and incubated at 37 °C. After 4h, the absorbance at 570 nm was measured. To confirm the MTT assay results, the fluorescent live-cell Promega ApoLive-Glo Multiplex protease activity assay kit was also used to measure the viability of WT fibroblasts, following the manufacturer's instruction.

### RNA extraction and RT-PCR

RNA extraction and RT-PCR was performed as previously described (8). In brief, using the RNeasy Plus Mini Kit (Qiagen) and High-Capacity RNA-to-cDNA Kit (Thermo Fisher Scientific), RNA was extracted from HDQ-P1 cells and cDNA was generated. Quantitative real-time RT-PCR for detection of *TP53* (5′ primer: CGCTTCGAGATGTTCCGAGA; 3′ primer: CTTCAGGTGGCTGGAGTGAG), *ETF1* (5′ primer: GCGGATTGCAACACATGCAG; 3′ primer: TTCTCGCCTCCTCCTCCCTAA), *UPF1* (5′ primer: GGGACCTGGGCCTTAACAAG; 3′ primer: ATGAGCCGCATGTCAGAGTC), and *SMG1* (5′ primer: GACTGGCAACCCAGAACTGA; 3′ primer: CCGAGACACCACAGCTGAAT) mRNAs was performed using the ABI StepOnePlus Real-Time PCR system.

### Statistical analysis

Statistical analysis was performed using GraphPad Prism 8.0. Data on the graphs were presented as mean ± SD. Two-way analysis of variance (ANOVA) was used to analyze the difference between different treatments and differences were considered significant at a *P*-value of <0.01.

## RESULTS

### Effect of siRNA-mediated knockdown of *eRF3a* and *eRF3b* on PTC readthrough

**Figure 1.**
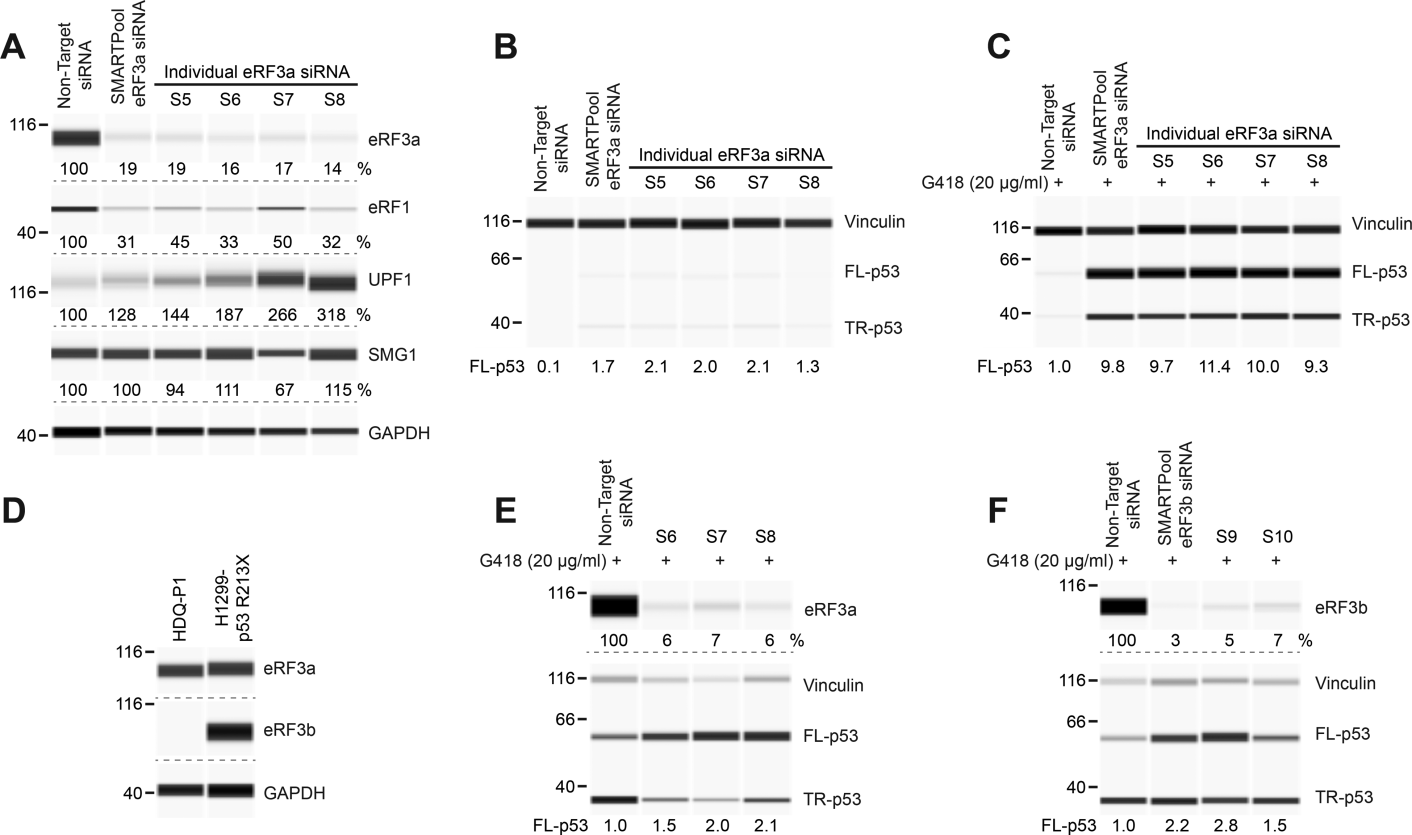
Effect of siRNA knockdown of *eRF3a* and *eRF3b* on *TP53* PTC readthrough. (**A**) HDQ-P1 cells with a homozygous nonsense mutation in the *TP53* gene (R213X) were transfected with either non-target siRNA or several ON-TARGETplus siRNAs targeting *eRF3a*. Levels of SURF complex components eRF3a, eRF1, UPF1 and SMG1 were measured 96 h after transfection. GAPDH was used as loading control. (**B**) p53 levels (full-length, FL-p53; truncated, TR-p53) were measured in the same extracts as in **A**. Vinculin was used as loading control. (**C**) p53 and vinculin levels were measured in transfected cells exposed to 20 μg/ml G418 for 48 h. (**D**) eRF3a and eRF3b levels were measured in HDQ-P1 and H1299-p53 R213X cell extracts. (**E, F**) H1299-p53 R213X cells were transfected with non-target siRNA or several ON-TARGETplus siRNAs targeting *eRF3a* (**E**) or *eRF3b* (**F**). eRF3a and eRF3b levels were measured 96 h after transfection. 48 h after transfection, samples were exposed to 20 μg/ml G418 for another 48 h and p53 and vinculin levels were determined. Protein levels in all panels were measured using automated capillary electrophoresis western analysis. In this and subsequent figures, equal amounts of protein lysate were loaded in all capillaries in each panel, except where indicated. The amounts of eRF3a, eRF3b, eRF1, UPF1 and SMG1 are expressed as percentage of levels in untreated cells. As FL-p53 is essentially undetectable in untreated HDQ-P1 cells in most experiments, FL-p53 levels are expressed relative to the amount of FL-p53 in cells treated with 20 μg/ml G418. Dashed lines indicate different capillaries.

Previous studies have shown that depletion of *eRF3* in yeast and siRNA knockdown of *eRF3a* in human cell lines promotes PTC readthrough in reporter assays (16,19). To examine whether down-regulating eRF3a can also induce the PTC readthrough of an endogenous gene in human cells, we selected the mammary carcinoma HDQ-P1 cell line with homozygous nonsense R213X mutation in the *TP53* gene. This mutation was previously shown to be responsive to PTC readthrough treatments (8,30). Transfection of different siRNAs targeting *eRF3a* reduced eRF3a protein levels to 14–19% of non-target siRNA controls, as measured by automated capillary western analysis (Figure 1A). To find out whether down-regulation of eRF3a would induce PTC readthrough we measured p53 levels. Cells transfected with non-target siRNA expressed essentially no detectable full-length p53, the readthrough product (Figure 1B). Transfection with *eRF3a* siRNAs induced the production of full-length p53, to about twice the level induced by exposure to G418 at 20 μg/ml, a suboptimal concentration for readthrough (Figure 1B and C). ASOs targeting *Gspt1* have previously been shown to promote PTC readthrough by G418 in a hemophilia mouse model (20). We reasoned that siRNA knockdown of *eRF3a* might also potentiate PTC readthrough by G418 in HDQ-P1 cells. Indeed, *eRF3a* knockdown strongly enhanced readthrough by 20 μg/ml G418, with 9 to 11-fold increase in full-length p53 compared to G418 alone (Figure 1C).

Human eRF3a and eRF3b can both bind eRF1 and function as translation termination factors. We were curious to also examine the effect of down-regulation of eRF3b on PTC readthrough. We determined that unlike eRF3a, eRF3b is barely expressed in HDQ-P1 cells (Figure 1D). Therefore, we carried out the experiment on p53-null H1299 cells stably transfected with p53-R213X, which express eRF3a as well as eRF3b (Figure 1D). Similar to HDQ-P1 cells, siRNAs targeting *eRF3a* in H1299 cells led to strong down-regulation of eRF3a and enhanced p53 readthrough by G418 ∼ 2-fold compared to G418 alone (Figure 1E). siRNA targeting of *eRF3b* resulted in >90% eRF3b down-regulation and combination with G418 increased production of full-length p53 up to 2.8-fold relative to G418 alone (Figure 1F). Taken together, these results indicate that knockdown of *eRF3a* or *eRF3b* in combination with low concentration of G418 can promote PTC readthrough in human cell lines.

It has been reported that in HEK293 cells, siRNAs targeting *eRF3a* not only reduced eRF3a levels but also induced the degradation of eRF1 (19). However, ASO-mediated depletion of *Gspt1* in another study using a hemophilia mouse model with nonsense mutations was only associated with minor reduction in eRF1 levels (20). To better understand the effects of eRF3a downregulation on the translation termination complex, we also measured eRF1 levels in HDQ-P1 cells. The different siRNAs targeting *eRF3a* all reduced eRF1 levels (Figure 1A). In addition to their role in translation termination, eRF1 and eRF3 also participate in the assembly of the SURF complex, which is formed on ribosomes stalled at PTCs, and contributes to NMD. Therefore, we also measured the effect of *eRF3a* siRNAs on other SURF complex proteins UPF1 and SMG1. *eRF3a* knockdown had no major effect on SMG1 levels but it induced an increase in UPF1 levels (Figure 1A). A previously described NMD autoregulatory feedback (31–33) may be responsible for UPF1 upregulation following downregulation of eRF3a and eRF1. Altogether, these results raise the possibility that the enhancement of PTC readthrough caused by *eRF3a* knockdown may be a consequence of disruption of both translation termination and NMD.

### Effect of small molecule eRF3 degraders on eRF3a, eRF1, UPF1 and SMG1

**Figure 2.**
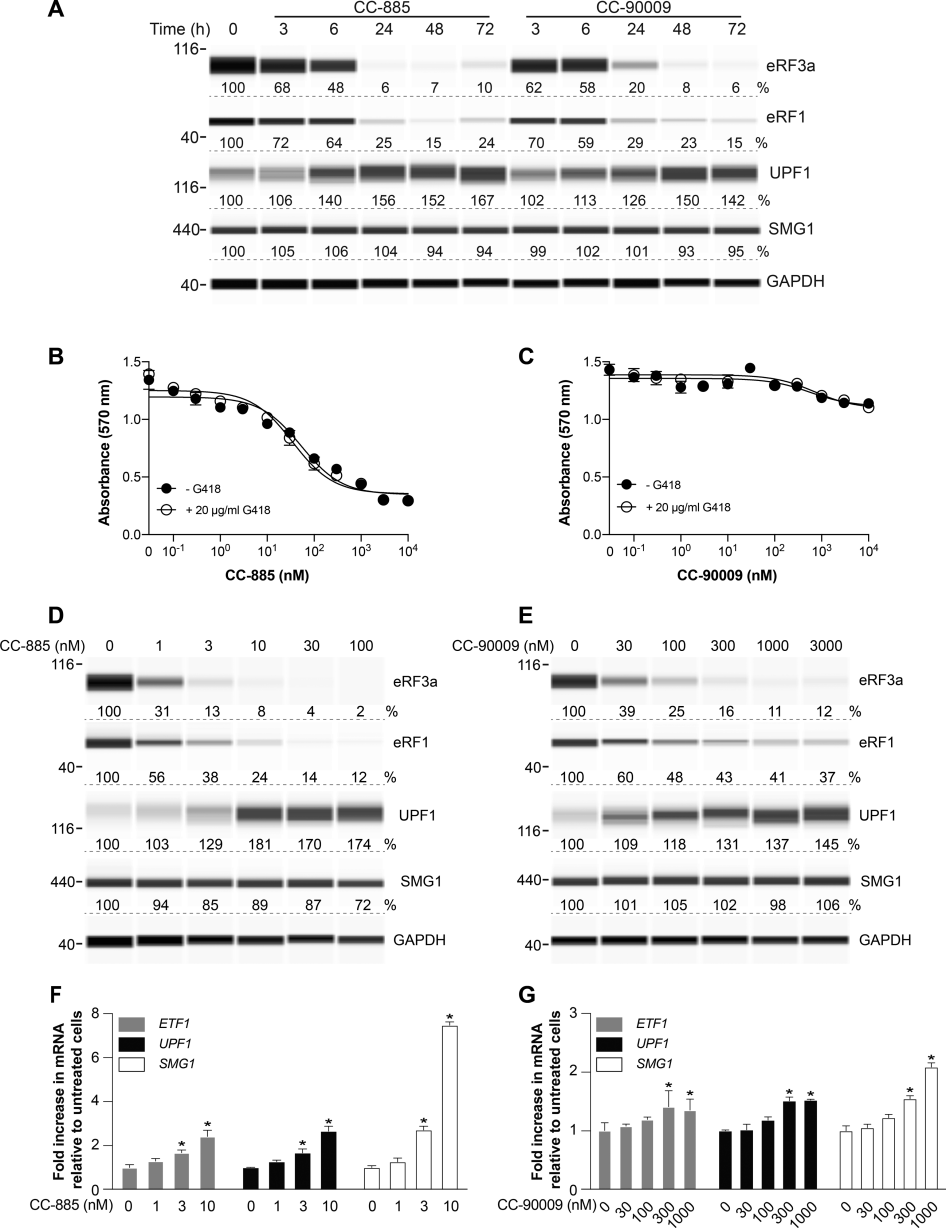
Effect of CC-885 and CC-90009 on SURF complex protein levels. (**A**) HDQ-P1 cells were exposed to 10 nM CC-885 or 1 μM CC-90009 for the indicated times and levels of eRF3a, eRF1, UPF1 and SMG1 were measured. GAPDH was used as loading control. (**B**, **C**) HDQ-P1 cells were exposed to different concentrations of CC-885 or CC-90009 for 48 h and cell viability was measured using the MTT assay in triplicate samples (±S.D.). (**D**, **E**) eRF3a, eRF1, UPF1 and SMG1 levels were measured. GAPDH was used as loading control. (**F**, **G**) *ETF1*, *UPF1* and *SMG1* mRNA levels were measured in triplicate samples (±S.D.). * indicates statistically significant difference to untreated samples (*P* < 0.01).

CC-885 and CC-90009 have recently been identified as ‘molecular glue’ compounds that co-opt the CUL4-DDB1-CRBN-RBX1 (CRL4^CRBN^) E3 ubiquitin ligase complex to trigger the ubiquitination and proteasomal degradation of eRF3a in AML cell lines (22,24). Given the ease of use of small molecules compared to gene knockdown technology, we next examined the effect of these compounds on eRF3a in HDQ-P1 cells. Cells were exposed to 10 nM CC-885 or 1 μM CC-90009 over the course of 72 h. Both compounds efficiently reduced eRF3a levels starting a few hours after compound addition and reaching > 90% reduction within 24 h for CC-885 and 48 h for CC-90009 (Figure 2A). These findings indicate that CC-885 and CC-90009 are capable of rapid and efficient degradation of eRF3a in cell types other than AML cell lines. To compare the effects of siRNAs targeting *eRF3a* to those of CC-885 and CC-90009 on other components of the SURF complex, we also measured the levels of eRF1, UPF1 and SMG1. Both compounds reduced eRF1 levels to ∼25% of controls, with a similar time dependence to eRF3a degradation (Figure 2A). Interestingly, both compounds also caused an increase in UPF1, again with a similar time dependence, and no major change in SMG1 levels (Figure 2A). The similarity of the effects of CC-885 and CC-90009 to those of *eRF3a* siRNAs provides strong evidence that their effects on eRF1 and UPF1 levels are a consequence of reduced eRF3a levels rather than off-target effects.

CC-885 and CC-90009 induce potent tumoricidal activity against AML cells (22–27) and a recent study using transcriptome and translatome analysis has shown that downregulation of eRF3a is associated with altered expression of many genes (34). Since CC-885 and CC-90009 considerably reduce the levels of eRF3a and eRF1 in HDQ-P1 cells, we next wished to examine their effects on cell viability. Exposure to increasing concentrations of CC-885 ranging from 0.1 nM to 10 μM for 48 h reduced cell viability with half-maximal effect at 52 nM (Figure 2B). Unlike CC-885, CC-90009 minimally affected HDQ-P1 cell viability at concentrations up to 10 μM (Figure 2C). Interestingly, G418, when used alone at 20 μg/ml, did not affect cellular viability and combination with CC-885 or CC-90009 did not further decrease cellular viability (Figure 2B and C). The unexpected low toxicity of CC-90009 suggests that selective downregulation of translation termination factors is tolerated by HDQ-P1 cells.

Next, we measured the effect of increasing concentrations of CC-885 and CC-90009 on SURF complex proteins eRF3a, eRF1, UPF1 and SMG1. CC-885 reduced eRF3a half-maximally at a concentration below 1 nM (Figure 2D). CC-885 also decreased eRF1 levels and increased UPF1 levels with a similar concentration dependence (Figure 2D). Similarly, CC-90009 caused a concentration-dependent decrease of eRF3a and eRF1 levels and increase in UPF1 (Figure 2E). Interestingly, high concentrations of CC-90009 did not induce as profound degradation of eRF3a and eRF1 as CC-885, with levels plateauing at ∼10% and ∼40% of untreated, respectively (Figure 2E). Neither compound had major effect on SMG1 levels (Figure 2D and E).

We were curious to examine whether changes in the expression of *ETF1* and *UPF1* might account for the changes in eRF1 and UPF1 protein levels caused by exposure to CC-885 and CC-90009. mRNA measurements showed CC-885 induced a moderate concentration-dependent increase in levels of *ETF1* and *UPF1* mRNAs, and a larger increase in *SMG1* mRNA, whereas CC-90009 caused smaller increases (Figure 2F and G). Therefore, decreased gene expression cannot explain the reduction in eRF1 levels caused by CC-885 and CC-90009, whereas increased *UPF1* mRNA is consistent with its increased cellular levels. A general increase in mRNA levels of components of the SURF complex is in agreement with NMD autoregulatory feedback (31–33).

### Effect of CC-885 and CC-90009 on PTC readthrough in HDQ-P1 cells

**Figure 3.**
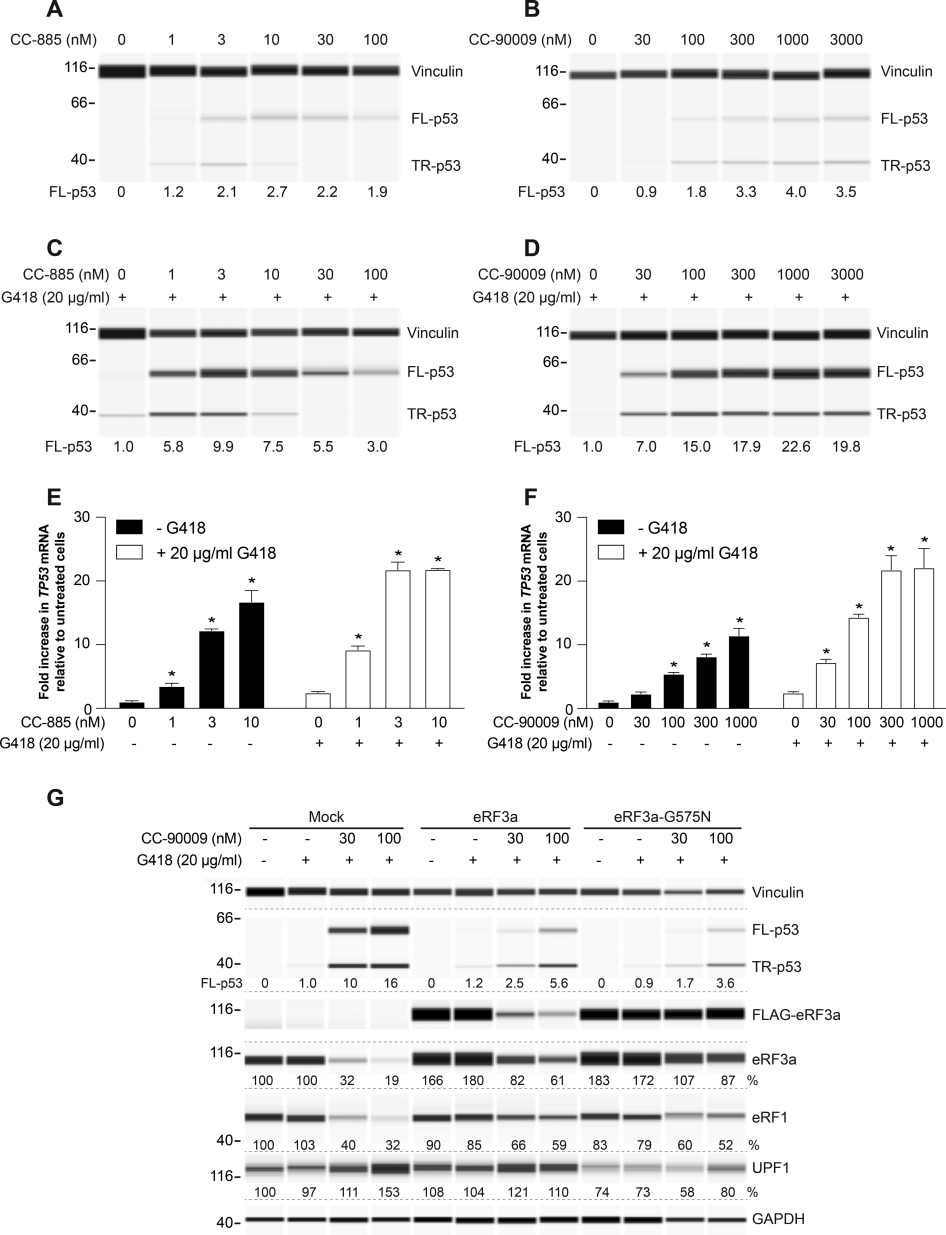
Effect of CC-885 and CC-90009 on *TP53* PTC readthrough. (**A, B**) HDQ-P1 cells were exposed to the indicated concentrations of CC-885 (**A**) or CC-90009 (**B**) for 48 h and p53 levels were determined. Vinculin was used as loading control. (**C, D**) HDQ-P1 cells were exposed to the indicated concentrations of CC-885 (**C**) or CC-90009 (**D**) in combination with 20 μg/ml G418 for 48 h and p53 levels were measured. Vinculin was used as loading control. (**E, F**) HDQ-P1 cells were exposed to the indicated concentrations of CC-885 (**E**) or CC-90009 (**F**) without or with 20 μg/ml G418 for 48 h and *TP53* level was measured in triplicate samples (±S.D.). (**G**) HDQ-P1 cells were transiently transfected with transfection reagents only (Mock), FLAG-tagged *eRF3a* or *eRF3a-*G575N constructs and exposed to the indicated concentrations of CC-90009 in combination with 20 μg/ml G418 for 48 h and p53, eRF3a, eRF1 and UPF1 were measured. Vinculin and GAPDH were used as loading controls. * indicates statistically significant difference to untreated samples (*P* < 0.01).

Next, we tested the effect of the compounds on PTC readthrough in HDQ-P1 cells. CC-885 and CC-90009 induced up to 2.7- and 4-fold increase in p53 readthrough, respectively, relative to 20 μg/ml G418 alone (Figure 3A and B). Combining CC-885 with G418 caused a strong increase in PTC readthrough with a maximum of ∼10-fold relative to G418 alone observed at 3 nM CC-885 (Figure 3C). The dose response was bell-shaped, with reduced PTC readthrough at higher CC-885 concentrations, perhaps due to cellular toxicity. However, unlike p53, vinculin levels did not significantly change at higher CC-885 concentrations, perhaps due to its longer half-life. The combination of CC-90009 and G418 resulted in a concentration-dependent increase in full-length p53, which reached levels ∼20-fold higher than those elicited by 20 μg/ml G418 alone and, unlike CC-885, plateaued at concentrations 100 nM and above (Figure 3D).

Since eRF1 and eRF3 translation termination factors also participate in modulating NMD, we next examined the effects of CC-885 and CC-90009 on R213X *TP53* mRNA, which is maintained at a low level in HDQ-P1 cells. Both compounds caused large concentration-dependent increases in *TP53* mRNA levels (Figure 3E and F), consistent with suppression of *TP53* mRNA degradation by NMD. Combining CC-885 and CC-90009 with G418 further increased *TP53* mRNA levels, in agreement with previous reports that increased readthrough reduced the recognition of mRNAs bearing nonsense mutations by the NMD machinery (8). These results indicate that CC-885 and CC-90009 can promote PTC readthrough by reducing translation termination as well as suppression of NMD. Although less potent than CC-885, CC-90009 showed a better activity window, being less toxic to HDQ-P1 cells at concentrations that strongly reduced eRF3a and eRF1 levels and strongly increased readthrough.

### Rescue of CC-90009 effects by eRF3-G575N

The *eRF3a* G575N mutation confers resistance to CC-90009-induced degradation (35). Overexpression of FLAG-tagged *eRF3a-*G575N in HDQ-P1 cells led to ∼80% increase in total (FLAG-tagged + endogenous) eRF3a protein levels compared to mock (transfection reagents only) (Figure 3G). eRF3a*-*G575N was not degraded during exposure to CC-90009 and G418 (Figure 3G), whereas endogenous eRF3a was, as shown by a decrease in total eRF3a. Overexpression of eRF3a-G575N considerably diminished the effects of the combination of G418 and CC-90009 on PTC readthrough, eRF1 and UPF1. Specifically, enhancement of PTC readthrough was reduced from 10-fold to 1.7-fold at 30 nM CC-90009 and from 16-fold to 3.6-fold at 100 nM CC-90009, compared to mock; eRF1 was not degraded as profoundly and UPF1 was not upregulated (Figure 3G). By comparison, overexpression of FLAG-tagged eRF3a led to a ∼60% increase in total eRF3a and FLAG-tagged eRF3a was degraded during exposure to CC-90009 and G418 (Figure 3G). However, total eRF3a degradation was not as profound as in mock. This was not unexpected as increasing the level of expression of the molecular target of a drug can often lead to increased drug resistance (36–38). In these cells, readthrough, eRF1 and UPF1 levels were intermediate between eRF3a-G575N and mock (Figure 3G). Overexpression of eRF3b caused a similar effect (Supplementary Figure S1). These results illustrate the central role of eRF3 degradation in the effects of CC-90009 on PTC readthrough and on the levels of SURF complex components.

### Effect of CC-885 and CC-90009 on readthrough of TAG and TAA nonsense mutations

**Figure 4.**
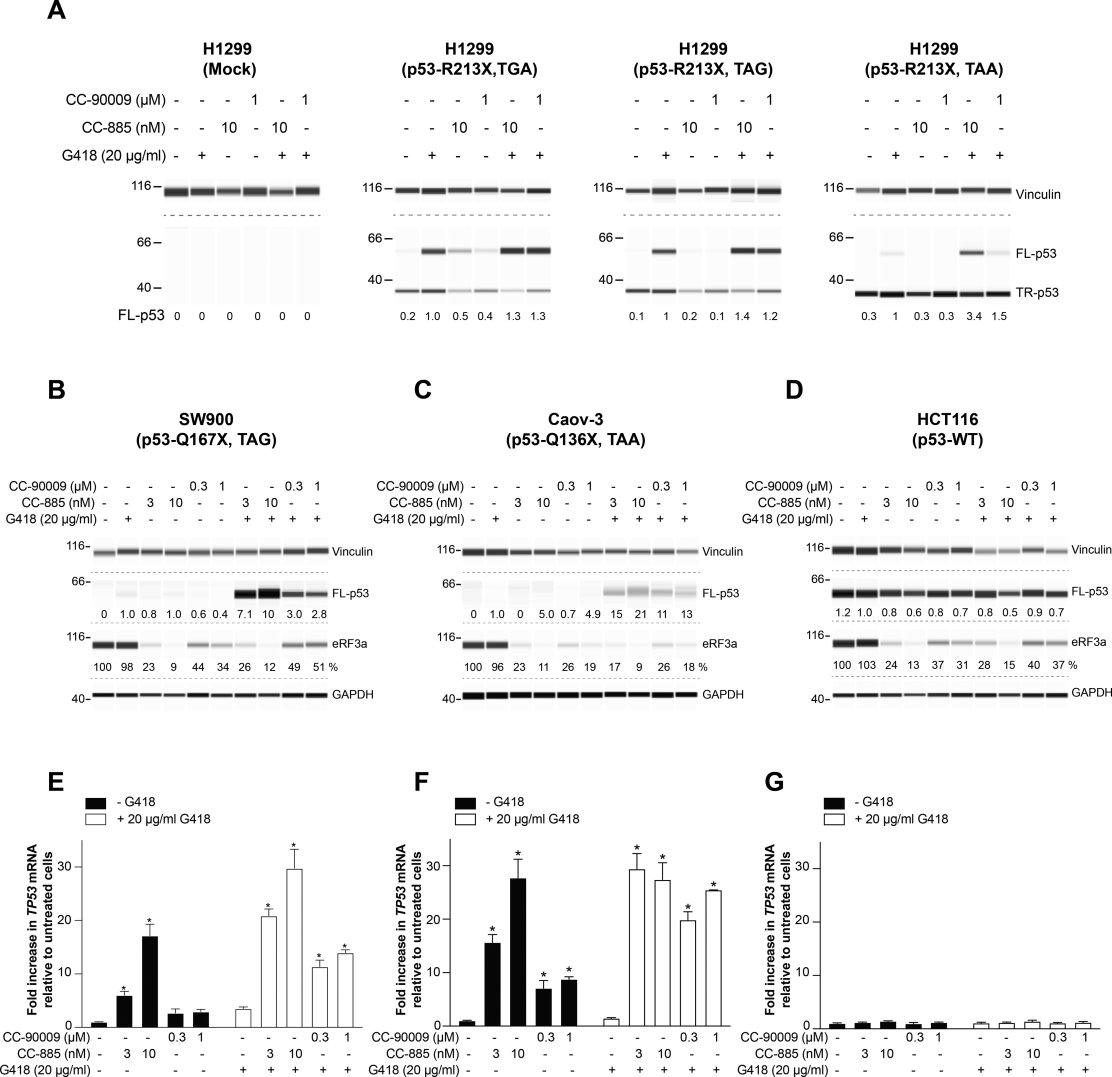
Effect of CC-885 and CC-90009 on TGA, TAG and TAA nonsense mutations. (**A**) H1299 cells transiently transfected with the indicated constructs were exposed for 48 h to 10 nM CC-885 or 1μM CC-90009 alone or in combination with 20 μg/ml G418 and p53 levels were measured. Vinculin was used as loading control. Mock: transfection reagents only. (**B–D**) SW900 (**B**), Caov-3 (**C**) and HCT116 (**D**) cells were exposed to the indicated concentrations of CC-885 or CC-90009 in combination with 20 μg/ml G418 for 48 h and p53 and eRF3a levels were measured. Vinculin and GAPDH were used as loading controls. Longer exposures are presented for the TAA mutations in panels **A** and **C** to better illustrate the lower readthrough levels. (**E–G**) SW900 (**E**), Caov-3 (**F**), and HCT116 (**G**) cells were exposed to the indicated concentrations of CC-885 or CC-90009 without or with 20 μg/ml G418 for 48 h and *TP53* level was measured in triplicate samples (± S.D.). * indicates statistically significant difference to untreated samples (*P* < 0.01).

Combining G418 with CC-885 or CC-90009 induces strong readthrough of a *TP53* TGA nonsense mutation in HDQ-P1 cells. TGA is known to be the leakiest termination codon and the easiest PTC to induce readthrough (39–41). We next examined readthrough of the intermediate (TAG) and the most stringent (TAA) termination codons. To compare the effect of the compounds on all three PTCs in the same gene and at the same position, p53-null H1299 cells were transiently transfected with *TP53* cDNA constructs that contained TGA, TAG or TAA at codon 213. 48 h after transfection, the cells were exposed to the compounds for another 48 h and p53 readthrough was analyzed. We note that unlike cells such as HDQ-P1 with endogenous p53-R213X, these transfected cells express large amounts of truncated p53 and detectable full-length p53 (Figure 4A), likely because their *TP53* transcripts escape degradation by NMD as a result of lacking exon junction complexes. Also, G418 induced production of full-length p53 more efficiently at TGA and TAG than TAA (Figure 4A). Combination of G418 and CC-885 or CC-90009 only slightly enhanced TGA, TAG and TAA readthrough over G418 alone and the overall readthrough remained considerably lower for TAA than TGA and TAG (Figure 4A). This low readthrough activity in cells transfected with p53-R213X cDNA constructs is reminiscent of the low PTC readthrough enhancement of siRNAs targeting *eRF3a* and *eRF3b* in H1299-p53 R213X cells (Figure 1E and F). As mentioned earlier, CC-885 and CC-90009 contribute to PTC readthrough by both reducing translation termination efficiency and suppressing NMD. It is likely that the lack of NMD degradation of *TP53* transcripts generated from cDNA explains the higher basal readthrough and lower readthrough enhancement by CC-885 and CC-90009.

To address the shortcomings associated with transfected constructs, we also examined SW900 and Caov-3 cells with endogenous homozygous TAG and TAA *TP53* nonsense mutations, respectively, as well as HCT116 cells with WT *TP53*. CC-885 and CC-90009 reduced eRF3a levels in all three cell lines and in combination with 20 μg/ml G418 they considerably increased p53 readthrough over G418 alone at TAG and TAA but they had no effect on WT p53 (Figure 4B–D). However, the overall levels of readthrough observed at TAA remained very low. CC-885 caused a large concentration-dependent increase in *TP53* mRNA in both SW900 and Caov-3 cell lines whereas CC-90009 caused a smaller increase (Figure 4E and F). By contrast, CC-885 and CC-90009 did not affect WT *TP53* mRNA levels in HCT116 cells (Figure 4G). The combination of CC-885 or CC-90009 and G418 caused a large increase in *TP53* mRNA in the mutant *TP53* cell lines but not in the WT *TP53* cell line (Figure 4E–G), indicating robust NMD suppression. These results indicate that combining CC-885 and CC-90009 with G418 can induce readthrough at all three PTCs. However, PTC readthrough at TAA remained low despite the considerable increase in *TP53* mRNA levels, indicating that better readthrough compounds than G418 will be necessary to elicit robust readthrough at TAA nonsense mutations.

### PTC readthrough activity of CC-885 and CC-90009 in genetic disease models

In principle, small molecules that induce PTC readthrough could be used to treat rare genetic disorders caused by nonsense mutations. To exemplify whether eRF3 degraders alone or in combination with aminoglycosides have potential to be used as a PTC readthrough treatment, we tested CC-885 and CC-90009 in cells derived from four patients with different genetic diseases: primary fibroblasts from a patient with mucopolysaccharidosis type I-Hurler (MPS I-H) [α-l-iduronidase: p.W402X/W402X], primary fibroblasts from a patient with neuronal ceroid lipofuscinosis (CLN2) [TPP1 (tripeptidyl peptidase 1): p.R127X/R208X], a myoblast line from a patient with Duchenne muscular dystrophy (DMD) [Dystrophin: p.E2035X], and a keratinocyte line from a patient with junctional epidermolysis bullosa (JEB) [Collagen XVII: p.R688X/R688X].

**Figure 5.**
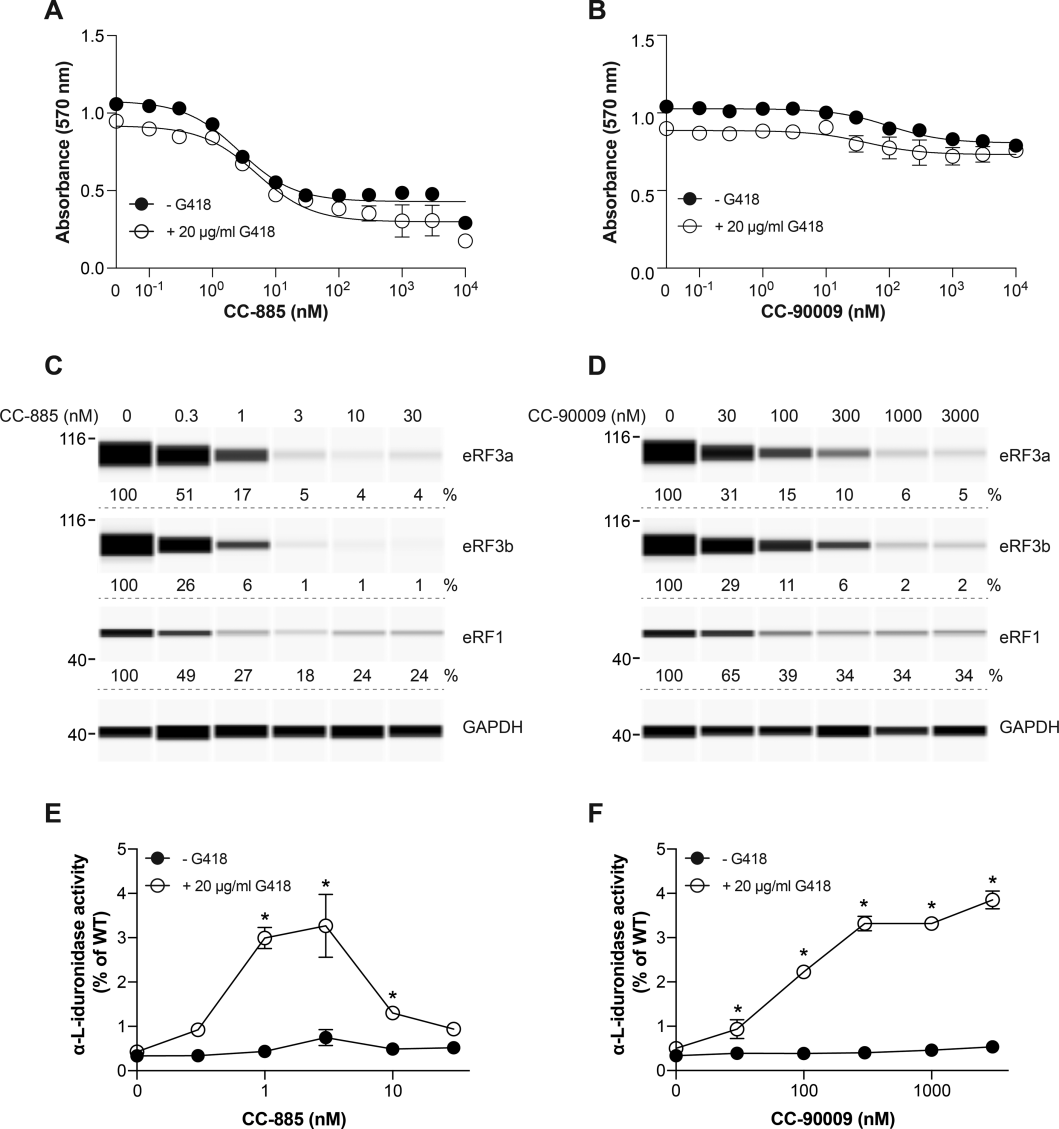
Effect of CC-885 and CC-90009 on *IDUA* PTC readthrough. (**A, B**) Fibroblasts derived from an unaffected individual were exposed to various concentrations of CC-885 (**A**) or CC-90009 (**B**) without or with 20 μg/ml G418 for 48 h and cell viability was measured using the MTT assay in triplicate samples (±SD, *n* = 3). (**C, D**) GM00798 primary fibroblasts derived from a MPS I-H patient with *IDUA* nonsense mutations (W402X/W402X) were exposed to the indicated concentrations of CC-885 (**C**) or CC-90009 (**D**) for 72 h and eRF3a, eRF3b and eRF1 levels were measured. GAPDH was used as loading control. (**E, F**) GM00798 fibroblasts were exposed to the same concentrations of CC-885 (**E**) and CC-90009 (**F**) as in **C** and **D**, with or without 20 μg/ml G418 for 72 h and α-l-iduronidase activity was measured in triplicate samples (±SD) and plotted as % of the activity measured in WT fibroblasts. * indicates statistically significant difference to untreated samples (*P* < 0.01).

**Figure 6.**
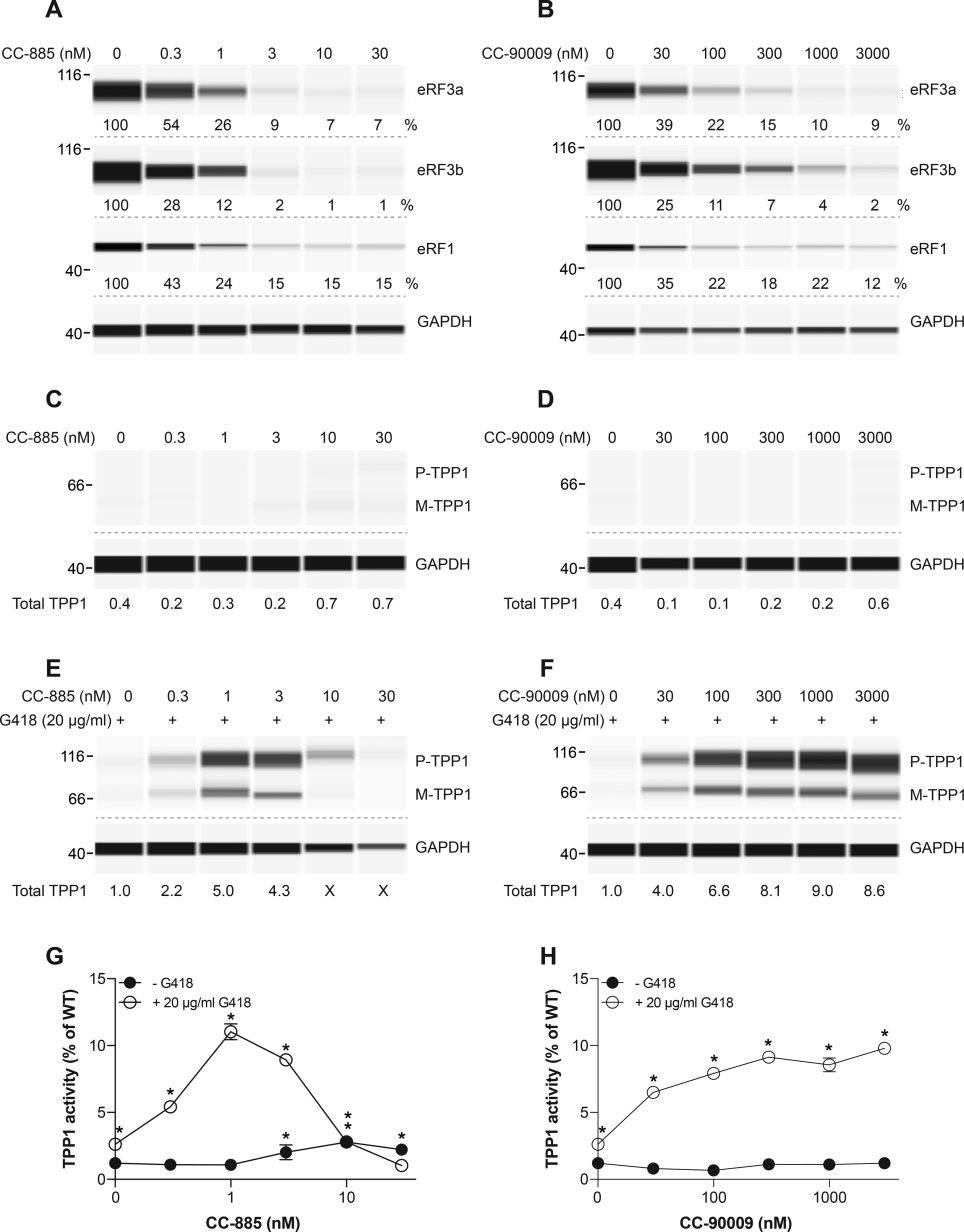
Effect of CC-885 and CC-90009 on *TPP1* PTC readthrough. (**A, B**) GM16485 primary fibroblasts derived from a late infantile neuronal ceroid lipofuscinosis (CLN2) patient with *TPP1* nonsense mutations (R127X/R208X) were exposed to the indicated concentrations of CC-885 (**A**) or CC-90009 (**B**) for 72 h and eRF3a, eRF3b and eRF1 levels were determined. (**C–F**) GM16485 fibroblasts were exposed to the indicated concentrations of CC-885 (**C**, **E**) or CC-90009 (**D**, **F**), alone (**C**, **D**) or in combination with 20 μg/ml G418 (**E**, **F**) for 72 h and TPP1 levels (proenzyme, P; mature, M) were measured. Total TPP1 levels (P + M) are expressed relative to the amount of total TPP1 in cells treated with 20 μg/ml G418. In panels **A–F**, GAPDH was used as loading control. In panel **E**, X indicates where lower amounts of protein were loaded because insufficient numbers of cells were recovered. (**G**, **H**) TPP1 activity from the same cell extracts as in **C–F** was determined in triplicate samples (±SD) and expressed as % activity relative to that of WT fibroblasts. * indicates statistically significant difference to untreated samples (*P* < 0.01).

**Figure 7.**
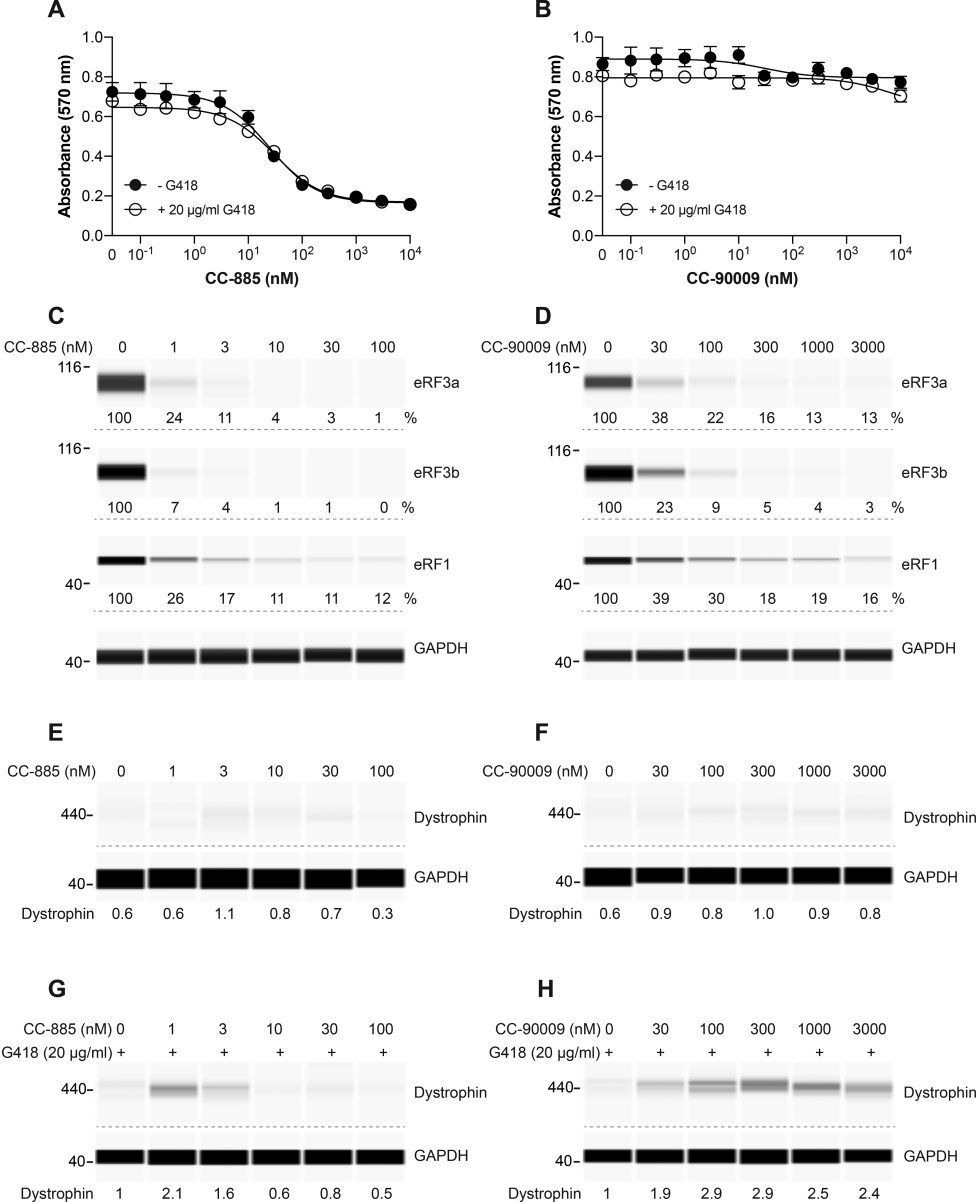
Effect of CC-885 and CC-90009 on *DMD* PTC readthrough. (**A, B**) Myoblasts derived from an unaffected individual were exposed to various concentrations of CC-885 (**A**) or CC-90009 (**B**) for 48 h and cell viability was measured using the MTT assay in triplicate samples (± S.D.). (**C, D**) HSK001 myoblasts derived from a DMD patient with a *DMD* nonsense mutation (E2035X) were differentiated into myotubes and exposed to the indicated concentrations of CC-885 (**C**) or CC-90009 (**D**) for 72 h and eRF3a, eRF3b and eRF1 levels were determined. (**E–H**) HSK001 myotubes were exposed to the indicated concentrations of CC-885 (**E**, **G**) or CC-90009 (**F**, **H**), alone (**E**, **F**) or in combination with 20 μg/ml G418 (**G**, **H**) for 72 h and dystrophin levels were determined. Dystrophin levels are expressed relative to the amount of dystrophin measured in samples treated with 20 μg/ml G418. GAPDH was used as loading control.

**Figure 8.**
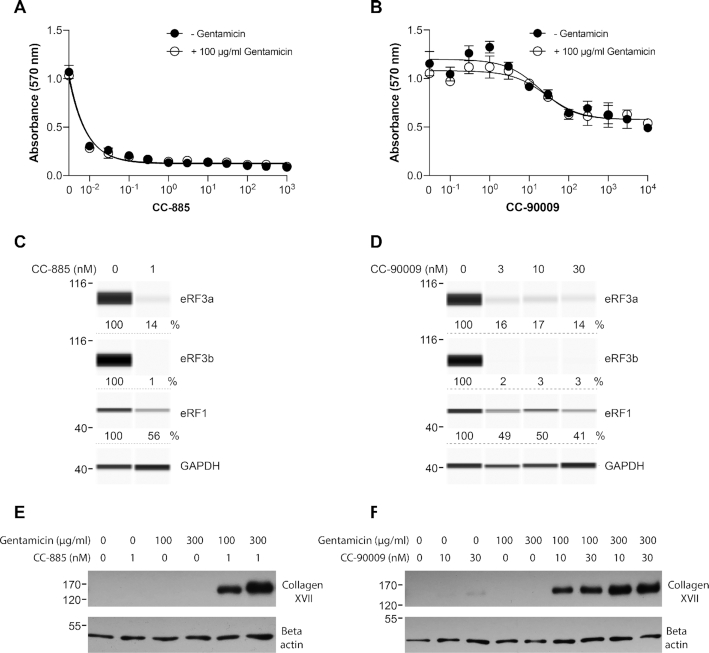
Effect of CC-885 and CC-90009 on *COL17A1* PTC readthrough. (**A, B**) HaCaT keratinocytes derived from an unaffected individual were exposed to various concentrations of CC-885 (**A**) or CC-90009 (**B**) with or without 100 μg/ml gentamicin sulfate for 48 h and cell viability was measured using the MTT assay in triplicate samples (±SD). Concentrations of CC-885 and CC-90009 are in nanomolar. (**C, D**) JEB01 keratinocytes derived from a JEB patient with a *COL17A1* nonsense mutation (R688X/R688X) were exposed to the indicated concentrations of CC-885 (**C**) or CC-90009 (**D**) for 72 h and eRF3a, eRF3b and eRF1 levels were determined using automated capillary electrophoresis western analysis. GAPDH was used as loading control. (**E, F**) JEB01 keratinocytes were exposed to the indicated concentrations of CC-885 (**E**) or CC-90009 (**F**) in the presence or absence of 100 or 300 μg/ml gentamicin sulfate for 72 h and Collagen XVII levels were measured by SDS-PAGE followed by traditional western blotting. *β*-actin was used as loading control.

Patient-derived cells and cells from unaffected individuals were exposed to increasing concentrations of eRF3 degraders ranging from 0.1 nM to 10 μM. Similar to HDQ-P1 cells, CC-885 was more toxic to all cells than CC-90009 (Figures 5A, 7A, 8A and Supplementary Figure S2). CC-885 was moderately toxic to fibroblasts from an unaffected individual (Figure 5A and Supplementary Figure S2) or MPS I-H patient (Supplementary Figure S2) but it strongly and potently reduced the viability of myoblasts and keratinocytes, with half-maximal effect at about 30 nM and less than 0.01 nM, respectively (Figures 7A and 8A). CC-90009 was not toxic to fibroblasts or myoblasts (Figures 5B, 7B and Supplementary Figure S2) and moderately toxic to keratinocytes (Figures 8B). Addition of 20 μg/ml G418 to fibroblasts or myoblasts, or 100 μg/ml gentamicin to keratinocytes did not further aggravate the cytotoxicity of CC-885 (Figures 5A, 7A, 8A and Supplementary Figure S2) or CC-90009 (Figures 5B, 7B, 8B and Supplementary Figure S2). CC-885 and CC-90009 caused a concentration-dependent decrease of eRF3a, eRF3b and eRF1 levels in all patient-derived cells tested (Figures 5C, D, 6A, B, 7C, D and 8C, D). Therefore, CC-885 and CC-90009 can deplete eRF3a, eRF3b and eRF1 in a variety of human cell types. Interestingly, at concentrations that caused more than 80% depletion of eRF3a and eRF3b, CC-90009 was much less toxic to all patient-derived cells than CC-885.

We next examined the effect of the compounds on PTC readthrough. The patient-derived cells were exposed to increasing concentrations of CC-885 or CC-90009 for 72 h without or with G418 or gentamicin. In MPS I-H fibroblasts, we observed negligible α-L-iduronidase activity in untreated cells or cells treated with CC-885, CC-90009 or 20 μg/ml G418 alone (Figure 5E and F). However, in combination with G418, CC-885 strongly increased α-l-iduronidase activity which peaked at over 3% of the enzyme activity seen in unaffected (WT) fibroblasts (Figure 5E). α-L-iduronidase activity decreased at CC-885 concentrations higher than 3 nM, perhaps due to cellular toxicity (Figure 5E). Remarkably, combining CC-90009 with G418 resulted in concentration-dependent increase in α-l-iduronidase activity higher than 3% of WT at concentrations above 100 nM CC-90009 (Figure 5F). This level would be expected to be sufficient for therapeutic results as it is known that patients with ∼1% residual α-L-iduronidase activity experience a milder form of the disease than patients with no detectable activity (42).

Untreated CLN2 fibroblasts displayed no detectable proenzyme or mature TPP1 in western analysis (Figure 6C and D). Exposure to CC-885 or CC-90009 induced barely detectable TPP1 production (Figure 6C and D). Exposure to G418 alone caused a small increase in TPP1 levels and a strong concentration-dependent increase in TPP1 production was observed when it was used in combination with eRF3 degraders (Figure 6E and F). Here again, TPP1 protein levels decreased at higher CC-885 concentrations but not at higher CC-90009 concentrations. The same extracts were also used to measure TPP1 enzyme activity. Consistent with western analysis data, no large increase in enzyme activity was observed in CLN2 fibroblasts treated with CC-885 or CC-90009 alone. Exposure to 20 μg/ml G418 caused a small increase in TPP1 activity that was strongly enhanced in combination with CC-885 (Figure 6G) or CC-90009 (Figure 6H) and reached a maximum of approximately 10% of that of unaffected fibroblasts. Similar to western analysis data, TPP1 enzyme activity diminished at CC-885 concentrations higher than 3 nM (Figure 6G), but plateaued at higher CC-90009 concentrations (Figure 6H). This level of restoration of enzyme activity, which occurred within 72 h exposure to compounds, might also be sufficient for therapeutic effect (43).

Untreated DMD myoblasts showed no detectable dystrophin (Figure 7E and F). Exposure to CC-885, CC-90009 or G418 alone induced a small amount of full-length dystrophin (Figure 7E-H). Again, the combination of G418 with eRF3 degraders strongly enhanced PTC readthrough, with CC-90009 displaying a larger activity window than CC-885 (Figure 7G and H).

Recent clinical studies report that systemic or topical gentamicin can induce PTC readthrough in patients with junctional or recessive dystrophic epidermolysis bullosa (44–47). Untreated JEB keratinocytes showed no detectable collagen XVII as measured by western blotting (Figure 8E and F). Exposure of these cells to 100–300 μg/ml gentamicin or 1 nM CC-885 did not increase collagen XVII levels while exposure to 10 and 30 nM CC-90009 resulted in a slight, barely detectable increase (Figure 8E and F). Combination of gentamicin with either CC-885 or CC-90009 clearly increased collagen XVII production (Figure 8E and F).

## DISCUSSION

This study shows that lowering the cellular levels of eRF3 is associated with a low level of PTC readthrough that can be considerably increased in combination with the readthrough aminoglycosides G418 or gentamicin. These results have implications for our understanding of the role eRF3 plays in PTC readthrough as well as the potential application of PTC readthrough to treatment of genetic diseases caused by nonsense mutations.

Consistent with a previous report (19), we observed that siRNAs targeting *eRF3a* led not only to downregulation of eRF3a protein but also to reduced levels of eRF1, the other component of the binary complex that mediates translation termination. One possible explanation for eRF1 reduction following eRF3a downregulation is that eRF1 might be proteolytically degraded when not complexed with eRF3a to adjust its level to that of the remaining eRF3a for the formation of the translation termination complexes, as proposed by others (19). eRF3a and eRF1 are also components of the SURF complex that forms on ribosomes stalled at PTCs and triggers degradation of the mRNA by NMD. siRNAs targeting *eRF3a* caused clear upregulation of the SURF complex component UPF1. It has previously been shown that knockdown of NMD factors such as UPF2, UPF3b, SMG1, SMG6 and SMG7 upregulates UPF1 and additional NMD factors, reflecting feedback regulation of NMD factors (31–33). A recent study analyzing the transcriptome and translatome of HCT116 cells upon UPF1 and eRF3a downregulation demonstrated increased expression of most NMD factors in UPF1-depleted cells, whereas UPF1 was not upregulated and only some NMD factors were upregulated in eRF3a-depleted cells (34). It is possible that the 80% reduction of eRF3a achieved in that study was not sufficient to upregulate UPF1 or that eRF3b expression in HCT116 cells compensated for lack of eRF3a. By contrast, in the HDQ-P1 cells used in the present study, *eRF3a* siRNAs clearly upregulated UPF1. This stronger effect may be due to the higher (>80%) downregulation of eRF3a achieved here or to the fact that HDQ-P1 cells express very low levels eRF3b.

The small molecules CC-885 and CC-90009 were previously shown to mediate the proteasome-dependent degradation of eRF3a in AML cells (22,24). We show that they also induce eRF3a degradation in several cancer cell lines as well as normal cells such as fibroblasts, keratinocytes and myoblasts and also induce the degradation of the closely related eRF3b. Interestingly, like *eRF3a* siRNA, CC-885 and CC-90009 also caused reduction of eRF1 and increase of UPF1 levels. The similarity of the effects of the siRNAs targeting *eRF3a* and the small molecule eRF3a degraders on eRF1 and UPF1 is strong evidence that they are consequences of the reduced eRF3 levels rather than off-target effects.

The suppression of NMD by CC-885 and CC-90009 inferred from observation of UPF1 upregulation is further substantiated by their effects on the levels of *TP53* mRNA bearing all three nonsense mutations in HDQ-P1, SW900 and Caov-3 cells (R213X-TGA, Q167X-TAG, and Q136X-TAA, respectively). Indeed, exposure to these compounds caused large increases in *TP53* mRNA in all three cell lines. Importantly, CC-885 and CC-90009 did not increase the levels of WT-*TP53* mRNA, which is not subject to NMD control. In principle, the increase in levels of *TP53* mRNA bearing nonsense mutations could be primarily due to suppression of NMD or it could be a consequence of enhanced PTC readthrough, which is known to shield mRNA from recognition by NMD (8,48,49). The observation that strong increases in *TP53* mRNA levels were observed in the presence of CC-885 and CC-90009 even in Caov-3 cells where readthrough was essentially undetectable because of the stringent TAA mutation argues for direct suppression of NMD rather than the consequence of PTC readthrough. In addition, when CC-885 and CC-90009 were combined with G418, which led to increased PTC readthrough, *TP53* mRNA levels rose further, likely as a result of the additional contribution of PTC readthrough to NMD suppression and therefore mRNA stabilization. Additionally, the increased levels of truncated p53 observed in HDQ-P1 cells after exposure to *eRF3a* siRNAs or eRF3a degraders is likely the consequence of abrogation of NMD and stabilization of *TP53* mRNA bearing a nonsense mutation. Further increase in truncated p53 (8,30,49–51) in the presence of G418 combined with eRF3a downregulation indicates further stabilization of *TP53* mRNA as a result of increased PTC readthrough. Together, these data indicate that the degradation of eRF3a and eRF3b by CC-885 and CC-90009 affects not only translation termination efficiency but also NMD.

The effects of *eRF3a* and *eRF3b* siRNAs on p53 PTC readthrough were also very similar to those of CC-885 and CC-90009. They elicited only a low level of PTC readthrough when used alone but readthrough was enhanced in the presence of G418. Consistently, CC-885 and CC-90009 considerably increased the levels of *TP53* mRNA bearing a nonsense mutation but this was clearly not sufficient to elicit strong PTC readthrough. Two major mechanisms contribute to maintaining PTC readthrough at a low level in cells. First, mRNAs bearing nonsense mutations are kept at a very low levels because of recognition and degradation by NMD. Second, the ribosome effectively accommodates the binding of release factors to PTCs at the A site while excluding the binding of near-cognate aminoacyl-tRNAs, thus strongly favouring termination over readthrough. Our results show that increasing the levels of a mRNA bearing a PTC combined with a ∼90% reduction of eRF3 and eRF1 is still insufficient to elicit strong readthrough. G418 alters the conformation of the ribosome A site to enable pairing of a near-cognate aminoacyl-tRNA to a PTC, however at non-toxic concentrations it barely induces PTC readthrough. Previous studies have also shown that combination of G418 with NMD inhibitor compounds such as amlexanox and NMDI14 can only slightly increase PTC readthrough over G418 alone (52,53), while in this study strong PTC readthrough is observed when CC-885 or CC-90009 is combined with G418. These results support a ‘triple blow’ model whereby strong PTC readthrough may be elicited when reduced levels of the translation termination factors, suppression of NMD and altered conformation of the ribosomal A site are achieved concurrently.

PTC readthrough has potential for treatment of genetic disorders caused by nonsense mutations. However, a major concern in developing PTC readthrough compounds is the lack of a therapeutic window as current readthrough candidate drugs tend to be toxic. We show that combination of CC-885 or CC-90009 with G418 can induce levels of TPP1 and iduronidase activity predicted to be sufficiently high for therapeutic benefit. The combinations also increased readthrough in DMD and JEB cells but functional assays will be needed before predictions of therapeutic activity can be made. Nonetheless, because dystrophin and collagen XVII have long half-lives (>1 month), relatively infrequent administration of readthrough combinations may be sufficient to reach therapeutic levels of readthrough proteins while minimizing toxicity. G418, although used here at low concentrations tolerated by cultured cells, is recognized as too toxic *in vivo*. Gentamicin, though less toxic and considerably less potent than G418, is also associated with oto- and nephrotoxicity when administered systemically in patients, although it is currently used successfully as a topical readthrough treatment in EB patients. ELX-02 is an aminoglycoside-like readthrough drug candidate currently in Phase 2 clinical trials in cystic fibrosis patients with nonsense mutations and its readthrough activity is also considerably enhanced by CC-90009 (Supplementary Figure S3). In this study, we observed severe cell toxicity by CC-885, which is consistent with its degradation of several CRBN neosubstrates (22). In contrast, CC-90009, which is in clinical trials for AML treatment and displays higher selectivity towards eRF3a (24), was not very toxic to various cell types tested in this study at concentrations that enhanced readthrough. Given that eRF3 controls not only translation termination and NMD but is also involved in other cellular pathways including cell cycle, mTOR signalling, apoptosis and mRNA deadenylation (12,54–57) and that *SUP35*, the gene encoding eRF3 is essential for yeast viability, it seems surprising that CC-90009 should show low toxicity to cultured human fibroblasts, keratinocytes, myoblasts and HDQ-P1 breast cancer cells. Moreover, transcriptome and translatome analysis of eRF3a-downregulated cells showed expression changes for many genes (34), although a recent proteomic study indicated selectivity of CC-90009 towards eRF3a with minimal to no effect on the rest of the proteome (24). However, we note that CC-90009 did not induce complete degradation of eRF3a, eRF3b or eRF1 at concentrations that strongly enhanced PTC readthrough. It is therefore possible that readthrough may be achieved at tolerable CC-90009 doses.

Since CC-885 and CC-90009 downregulate all three translation termination factors a potential concern is whether they would elicit readthrough at normal termination codons. Although not studied systematically, our examination of several proteins produced by PTC readthrough in the presence of CC-885 or CC-90009 in combination with G418 did not reveal any protein larger than full-length. This may be due to the existence of multiple in-frame stop codons in the 3′-UTR of most human genes, which would hinder efficient readthrough (51). Moreover, the intimate proximity of normal stop codons to the poly(A) tail also facilitates interaction of Poly(A)-binding protein and eRF3 to stimulate rapid translation termination (reviewed in ref.^48^). It was recently shown that when translational readthrough occurs over normal termination codons the 3′-UTR-encoded polypeptides promote aggregation of the C-terminally extended proteins and their targeting to lysosomes, which would also contribute to limiting the accumulation of C-terminally extended proteins (58). Based on these observations, we speculate that translational readthrough over normal termination codons is unlikely but further studies are required to address this issue.

The availability of drug candidates such as CC-90009 and ELX-02 that are already being tested in humans offers opportunities for faster exploration of their therapeutic potential for PTC readthrough in animal models of rare genetic diseases.

## Supplementary Material

gkab194_Supplemental_File
