# Supplementary material for: Effect of small molecule eRF3 degraders on premature termination codon readthrough
Source: Nucleic Acids Res. 2021 Mar 25;49(7):3692–708. doi: 10.1093/nar/gkab194 (PMC8053119; doi:10.1093/nar/gkab194)
Supplement: gkab194_Supplemental_File [file gkab194_supplemental_file.pdf]

Effect of small molecule eRF3 degraders on premature termination codon readthrough

Alireza Baradaran-Heravi[1], Aruna D. Balgi[1], Sara Hosseini-Farahabadi[1], Kunho Choi[1], Cristina Has[2] and Michel Roberge[1]

[1]Department of Biochemistry and Molecular Biology, University of British Columbia, Vancouver, BC, Canada V6T 1Z3

[2]Department of Dermatology, Medical Center-University of Freiburg, Faculty of Medicine, Freiburg, Germany

Corresponding author:
Michel Roberge
Dept Biochemistry & Molecular Biology
University of British Columbia
2350 Health Sciences Mall
Vancouver, BC, Canada, V6T1Z3
T. 604-652-9997
michelr@mail.ubc.ca

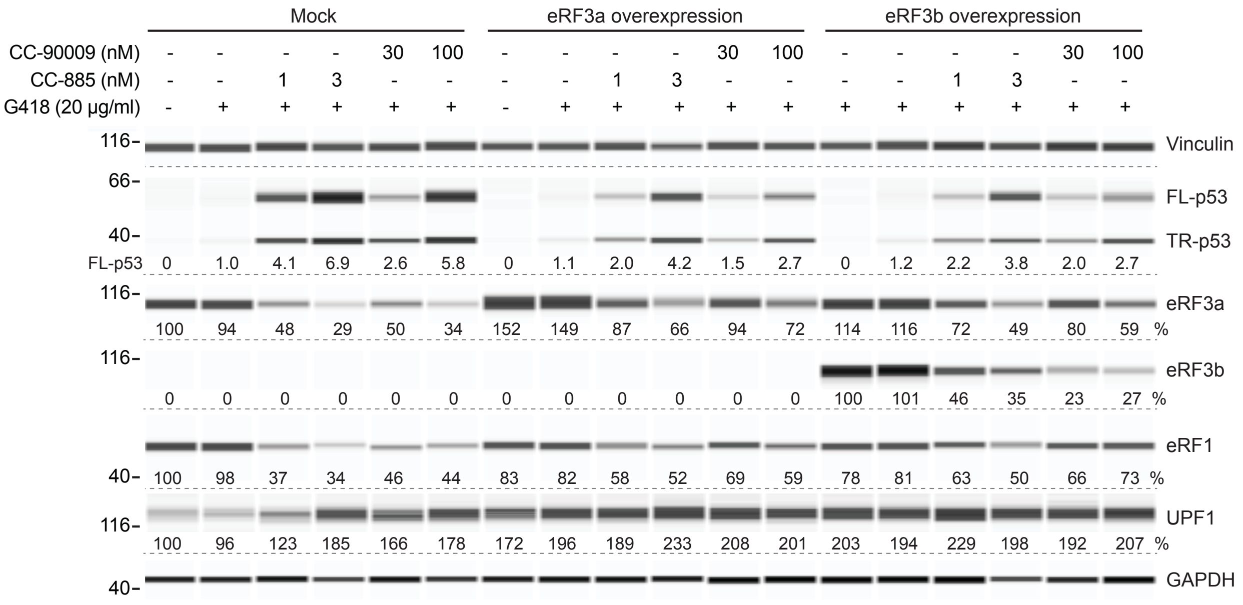

Supplementary Figure S1. Partial rescue of the effects of CC-885 and CC-90009. HDQ-P1 cells were transiently transfected with transfection reagents only (Mock), *eRF3a* or *eRF3b* constructs and exposed to the indicated concentrations of CC-885 or CC-90009 in combination with 20 µg/ml G418 for 48 h and p53, eRF3a, eRF3b, eRF1 and UPF1 were measured. Vinculin and GAPDH were used as loading controls.

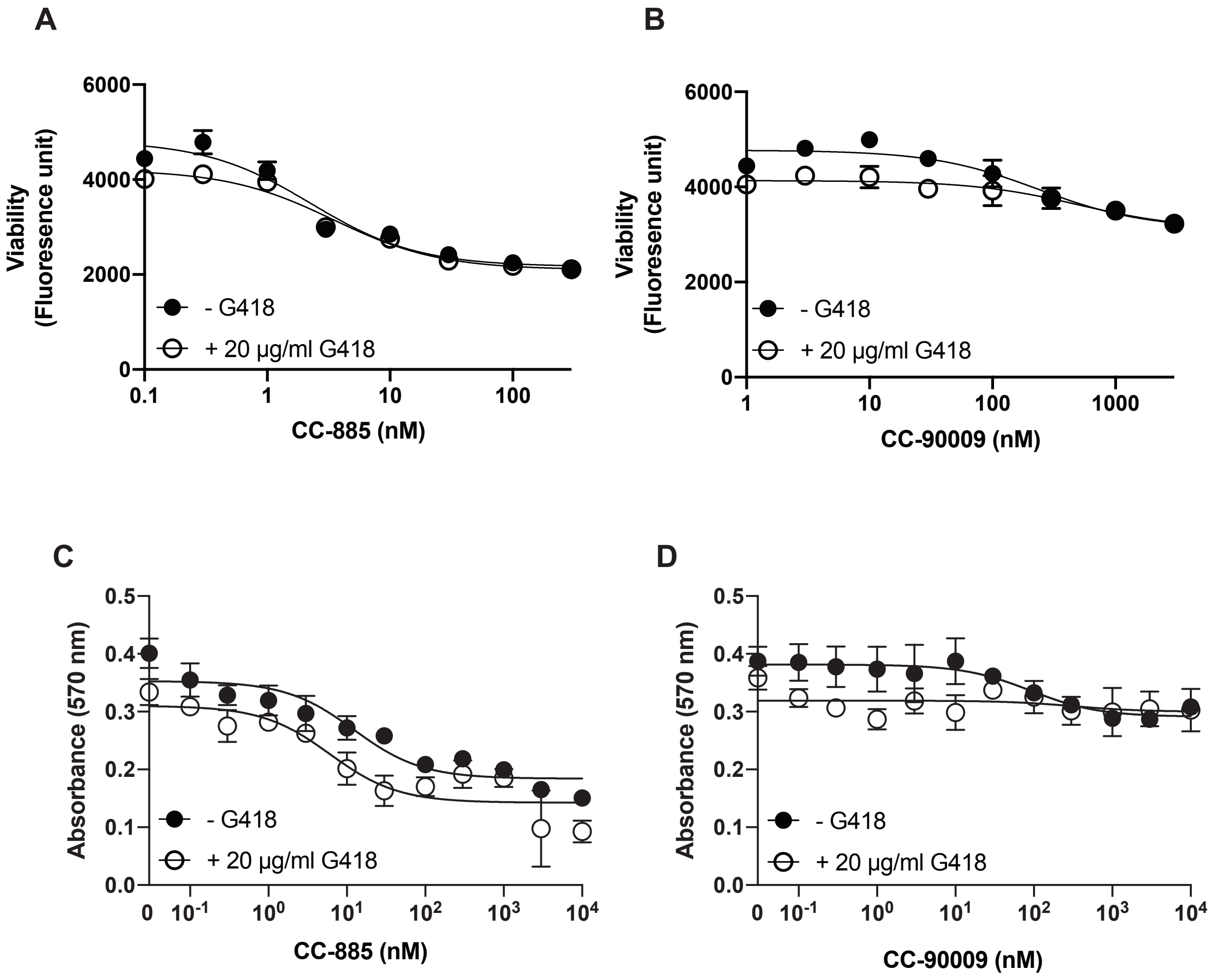

Supplementary Figure S2. Measurement of cell viability. Fibroblasts derived from an unaffected individual **(A**, **B)** or MPS I-H patient **(C**, **D)** were exposed to various concentrations of CC-885 **(A**, **C)** or CC-90009 **(B**, **D)** without or with 20 µg/ml G418 for 48 h and cell viability was measured using the Promega ApoLive-Glo Multiplex Assay kit **(A**, **B)** or the MTT assay **(C**, **D)** in triplicate samples (± S.D.).

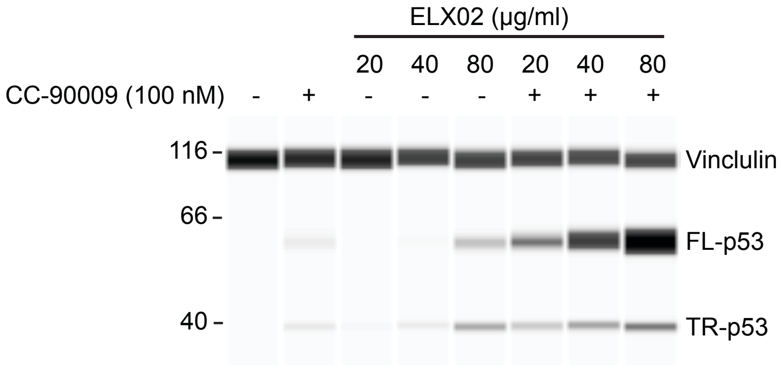

Supplementary Figure S3. Effect of CC-90009 on PTC readthrough by ELX-02.

HDQ-P1 cells were exposed to the indicated concentrations of ELX-02 with or without 100 nM CC-90009 for 48 h and p53 levels (full-length, FL-p53; truncated, TR-p53) were determined using automated capillary electrophoresis western analysis. Vinculin was used as loading control.
